# Process-Induced Crystal Surface Anisotropy and the Impact on the Powder Properties of Odanacatib

**DOI:** 10.3390/pharmaceutics16070883

**Published:** 2024-06-30

**Authors:** Isha Bade, Vikram Karde, Luke Schenck, Marina Solomos, Margaret Figus, Chienhung Chen, Stephanus Axnanda, Jerry Y. Y. Heng

**Affiliations:** 1 Department of Chemical Engineering, South Kensington Campus, Imperial College London, London SW7 2AZ, UK; isha.bade16@imperial.ac.uk (I.B.); v.karde@imperial.ac.uk (V.K.); 2 Oral Formulation Sciences, Merck & Co., Inc., Rahway, NJ 07065, USA; luke_schenck@merck.com (L.S.);; 3 Analytical Research & Development, Merck & Co., Inc., Rahway, NJ 07065, USA; margaret_figus@merck.com (M.F.); chien-hungbill.chen@merck.com (C.C.)

**Keywords:** particle properties, anisotropy, powder processing, crystalline API, characterization techniques

## Abstract

Crystalline active pharmaceutical ingredients with comparable size and surface area can demonstrate surface anisotropy induced during crystallization or downstream unit operations such as milling. To the extent that varying surface properties impacts bulk powder properties, the final drug product performance such as stability, dissolution rates, flowability, and dispersibility can be predicted by understanding surface properties such as surface chemistry, energetics, and wettability. Here, we investigate the surface properties of different batches of Odanacatib prepared through either jet milling or fast precipitation from various solvent systems, all of which meet the particle size specification established to ensure equivalent biopharmaceutical performance. This work highlights the use of orthogonal surface techniques such as Inverse Gas Chromatography (IGC), Brunauer–Emmett–Teller (BET) surface area, contact angle, and X-ray Photoelectron Spectroscopy (XPS) to demonstrate the effect of processing history on particle surface properties to explain differences in bulk powder properties.

## 1. Introduction

Surface properties, including surface chemistry, energetics, and wettability, are key performance indicators of important pharmaceutical materials’ quality attributes such as their stability, dissolution rate, flowability, and dispersibility [1]. The physiochemical properties of the surface govern the interparticle interactions within the pharmaceutical solids and, therefore, influence their bulk powder properties [2]. It is possible to modify the particle surface using wet or dry coating techniques to enhance the particle characteristics such as to reduce cohesion and improve flowability [3], improve content uniformity [4], and elevate overall powder processibility [5,6,7,8].

Almost 90% of APIs are crystalline in nature [9] and, since the presentation of molecules at the surface of a crystal is facet dependent, an appreciation for surface anisotropy is key when considering particle properties. This variation in surface chemistry reportedly demonstrates anisotropy in surface wettability, surface energetics, and optical, mechanical, and magnetic properties of crystals [10,11]. Hence, elucidating the fundamental surface properties of single crystalline particles can provide the ability to modulate bulk characteristics of the API.

Various studies have shown the influence of downstream unit operations such as milling, crystallization and solvent selection, co-precipitation, and blending on particle properties [12,13,14]. Solvent effects can significantly impact crystalline morphology, which controls crystal surfaces (varying preferred facets) and, later on, could impact powder properties, even if surface area and particle size of the powders are comparable [15]. Dry milling, a common particle size reduction technique employed in the pharmaceutical industry, has been shown to increase the surface area but also to vary the surface energy due to preferential breakage [16,17,18]. Milling is an energy-intensive process and may result in increased lattice disorder and, therefore, a reduction in the crystallinity of the sample [19,20]. On the other hand, common size enlargement techniques such as wet granulation have also been shown to alter particle properties. Blending pharmaceutical powders with a liquid binder to produce agglomerates results in better powder dissolution and tensile strength [21,22]. Other processes like mixing [23,24], drying [25,26], and tablet compaction [27,28] also lead to changes in the critical quality attributes (CQAs) of the final drug particle as well as their intermediary properties during the downstream processing [22].

Multiple techniques have been adopted over the years, which have proven reliable for analyzing the particle properties of pharmaceutical powders. Inverse Gas Chromatography (IGC) is a robust, prevailing technique for surface energy measurements of powders and is also capable of determining their surface energy heterogeneity using the Finite Dilution (FD–IGC) method [29,30,31]. This entails obtaining a unique energy profile for each solid sample surface at various test probe coverage ratios, which, in turn, can help assess the effect of powder processing and batch-to-batch variability, as well as predict powder behavior [31]. Generally, a combination of analytical techniques is used to support and reinforce key conclusions. Contact angle measurements are commonly implemented to study the wettability of the solid surface towards various probe liquids such as water, diiodomethane, or ethylene glycol [32,33]. The angle that the liquid droplet produces on the sample surface can then be used to estimate surface energetics using well-established empirical equations from the literature such as Young’s equation [33] or Owen–Wendt’s method [34]. The knowledge of surface hydrophobicity and hydrophilicity from the aforementioned measurements can also aid in deducing the probable surface interactions based on the chemistry of the molecule. These interactions can be validated if the surface chemistry of the solid sample is known, which can be measured using a powerful technique called X-ray Photoelectron Spectroscopy (XPS) that can analyze the surface at a depth of 10 nm over an area of up to 10 µm [35]. The bonds available for surface interaction will vary based on the facet presented, thereby impacting the wettability and surface energy of the sample. Literature references are available on the use of these characterization techniques for powdered samples compacted into a tablet form, as well as macroscopic single crystals [36,37].

Previously, we have reported that jet milled versus precipitated Odanacatib exhibits significantly different powder properties, solid-state properties, dissolution, and tablet content uniformity (CU) [38]. This work aims to demonstrate the effect of processing history on particle surface properties with the use of orthogonal techniques such as IGC, Brunauer–Emmett–Teller (BET) surface area, contact angle, and XPS to support observed differences in bulk powder properties. Odanacatib, a drug inhibiting the cathepsin K that is responsible for osteoporosis [39], has been used as the model compound for this study, where adjacent comparisons between samples from different processing backgrounds are drawn. This is an interesting model compound since biopharma models suggested that micronization was needed to achieve full bioavailability. Furthermore, bottom-up precipitation routes were identified from multiple solvent systems that could achieve comparable size as jet milled API, but, in theory, with different distributions of crystalline facets based on the processing solvent. Finally, the crystallization could be modified to deliver larger particles with very different morphology to interrogate if mechanical fracture planes (vs. attachment energy planes) carried through jet milling. This was to pressure test if jet milling truly normalized API PSD, that is, can any API feedstock, size, and morphology be milled to the target size and surface area, with the final milled material meeting specifications agnostic to the parent lot properties. Single crystals of Odanacatib were also grown and analyzed using XPS to study the potential influence of anisotropy on the bulk powder behavior. This work will establish the impact of powder properties and the performance indicators on the processed drug samples.

## 2. Materials and Methods

### 2.1. Materials

**Figure 1:**
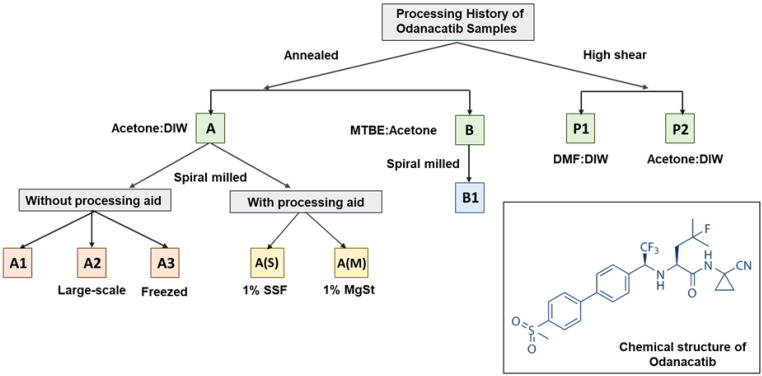
A flowchart depicting the processing route for different Odanacatib (MK-0822) samples, along with the chemical structure (bottom right).

Odanacatib samples with different processing histories (Figure 1) were acquired from Merck & Co., Inc. (Rahway, NJ, USA). A detailed description of the processing history of each sample can be found in the previous publication. Dimethyl sulfoxide (DMSO) (CAS: 67-68-5) of purity ≥ 99.9% was sourced from Sigma Aldrich and used as received.

Odanacatib samples were received as described in Table 1. Recrystallization in a 1:2 acetone–water mixed-solvent system generated sample A, with a larger PSD and rod-like morphology. This recrystallization process was analogous to pilot-scale recrystallization. In product development, recrystallized Odanacatib was subsequently jet milled, and, as such, sample A1 and sample A2 were acquired from Merck & Co., Inc, Rahway, NJ, USA and were representative of two unique sub-lots of jet milled API in storage. Sample A was spiral milled at the laboratory scale, and a sample was stored at −8 °C to prevent annealing (sample A3). Other subsets of this milled API were lubricated with 1% SSF (sample A(S)) and 1% MgSt (sample A(M)).

A unique morphology (high aspect ratio, needles) from that of sample A was prepared by recrystallization in 3:2 acetone–MTBE, generating sample B. Sample B was additionally spiral milled at the laboratory scale to generate sample B1. This was to explore if the jet milling process truly normalized differences in parent lots, or if the different aspect ratio of the starting material could result in breakage via preferred fracture planes that altered exposed crystals surfaces in the milled material.

Sample P1 and sample P2 were prepared from high-shear direct precipitation. A solution of Odanacatib was prepared in DMF and rapidly precipitated out in water, then seeded by sample A1 to encourage crystallization to sample P1. A similar approach generated sample P2 by precipitating a solution of Odanacatib in 9:1 acetone–water within a water antisolvent and again templating crystallization with sample A1 seed crystals. All as-received samples were of the same and only known crystalline form of Odanacatib.

### 2.2. Recrystallisation to Obtain Single Crystals

Single-crystal facet analysis was undertaken to relate crystal anisotropy with the distinct powder characteristics in different samples. Large single crystals were grown using slow solvent evaporation. The solubility of Odanacatib at room temperature in different solvents is as follows: DMSO > acetone > acetonitrile > ethanol > water. This was found by continuously mixing in known amounts of sample A1 in 5 mL of solvent at room temperature until the solution was saturated. To obtain seeds, the solution was allowed to settle and the supernatant was transferred to 10 mL vials. These were covered with parafilm that was perforated with a few 1 mm holes to allow for solvent evaporation at room temperature over time. After several days, crystals were obtained at the base of the vial, where, regardless of the solvent, high-aspect-ratio crystal habits were observed. Following the solvent screening process, due to DMSO providing the best balance between solubility, rate of evaporation and comparatively low-aspect-ratio crystals, allowing them to be easier to handle, it was chosen as the primary solvent for recrystallisation. Consequently, a saturated solution of DMSO and sample A1 was prepared and, following a period of 1 week of slow evaporation, seeds of 1–2 mm were obtained at the bottom of the vial, which were carefully isolated using blunt tweezers.

These single seeds were then individually placed in a 5 mL vial, topped up with the saturated solution, which was then allowed to evaporate in a similar manner as stated above in order for the seed to grow into a large single crystal. Larger crystals of approximately few millimeters were desired in order to perform XPS on the individual crystal facets. In general, it is unfavorable for needle crystals to grow as a standalone single crystal. Thus, it was unsurprising that the seed grew into a ‘stacked’ structure. It was assumed that the like facets would attach together [40] and, hence, the structure would exhibit the same external facets to those if it were a single crystal. This proved to be beneficial during the analysis of the crystals too, as the larger surface area provided by the stacked structure aided the XPS measurements.

### 2.3. Specific Surface Area Determination Using BET

#### 2.3.1. N_2_ Adsorption

Specific surface area (SSA) of each sample was obtained from analyzing low-temperature nitrogen adsorption isotherms (at 77 K), collected using a TriStar II analyzer (Micromeritics Instrument Corp., Norcross, GA, USA). Material was loaded into a sample tube and degassed under nitrogen at 35 °C for 1 h before analysis. After cooling to room temperature, the tube was weighed and placed into the adsorption port of the instrument. A static adsorption mode was used, including full equilibration after each adsorbate load. The adsorption isotherms were measured over a relative pressure, p/po, range of 0.001–0.995. The surface area was calculated via the Brunauer–Emmett–Teller (BET) method [41], using the relative pressure range from 0.10 to 0.30, where a linear isotherm is observed.

#### 2.3.2. IGC Octane Isotherm

The specific surface area of API samples was determined by Inverse Gas Chromatography using an IGC surface energy analyzer (IGC SEA, SMS, London, UK). About ~200–300 mg of material was packed into a salinized glass column (internal diameter = 3 mm) and plugged with salinized glass wool on both ends [29,30,31,42]. A jolting voltameter (Surface Measurement Systems, London, UK) was used to provide mechanical tapping to the sample to remove voids in the packed sample bed. The BET was analyzed using octane injections at 0%RH and 30 °C. Prior to BET measurement, the sample column was conditioned at the same conditions for a period of 2 h with helium as a carrier gas at 10 mL/min flow rate. Methane was used as a reference gas to determine the dead volume.

#### 2.3.3. Particle Size Distribution (PSD)

The particle size distribution (PSD) of each sample was obtained by using a laser-diffraction-based technique, Microtrac S3500. Material was loaded into a vial and 0.25% lecithin in isopar-G was added as a dispersion medium. The dispersed sample in lecithin/isopar-G solution was then introduced to the Microtrac sample cell. Microtrac measurement settings of irregular particles with particle refractive index of 1.51 and fluid refractive index of 1.42, 60% flow, and sonication power of 30 W were applied. Each sample was measured at 0 s, 30 s, 60 s, 90 s, and 120 s. Standard progression was applied in the analysis.

### 2.4. Surface Energy Heterogeneity Analysis

Packed sample columns prepared for IGC were subsequently conditioned at 30 °C and 0% Relative Humidity (RH) for 1 h under 10 mL·min^−1^ carrier gas (helium) flow rate prior to the measurement. Helium was used as a carrier gas at a flow rate of 10 mL·min^−1^ and methane was used as a reference gas to determine the dead volume. The analysis was carried out in the finite dilution range using a series of n-alkane probes (decane, nonane, octane, heptane, and hexane) to determine the dispersive interactions. Polar interactions were evaluated with ethyl acetate and dichloromethane as a monopolar base and monopolar acid, respectively. The surface energies were determined using the Schultz method with the peak com analysis approach [43].

### 2.5. Contact Angle Measurements

The processed API samples were analyzed for the wetting characteristics surface properties from contact angle measurements using advanced automated goniometer apparatus (Ramé-hart Instrument Co., Model 590, Succassuna, NJ, USA). The API tablets (6 mm diameter) were produced using a single-punch bench-top Gamlen tablet press (Gamlen Tableting, Heanor, UK) using a compaction load of 100 kg. The static contact angle was measured on each of the compacts in triplicate using de-ionized water as the test liquid at a drop volume of 10 µL. Additionally, to investigate the stability and stress relaxation behavior of the tablets, contact angle measurements were performed on the tablets stored for 1 day, 3 days, and 5 days.

### 2.6. X-ray Diffraction (PXRD)

#### 2.6.1. Powder X-ray Diffraction (PXRD)

Powder X-ray diffraction (PXRD) patterns of all Odanacatib samples were collected by a PANalytical X’Pert PRO X-ray diffractometer in reflection geometry using copper (Cu) Kαradiation (λ = 1.5405 Å, where λ is the wavelength) at 40 kV and 20 mA. The samples were in powder form and, hence, used as received, while the recrystallized single crystals were crushed using the back of a spatula to grind into fine powder. Approximately 5 mg of powder was placed on the sample holder and pressed into a thin layer. Measurements were conducted at a scanning rate of 50 s per step, with a step size of 0.013° over a diffraction angle range from 5° to 35°.

The experimental PXRD patterns were then compared with the powder diffraction pattern calculated in Mercury (version 2022.2.0, CCDC, Cambridge, UK) using the crystal structure data deposited in the Cambridge Structural Database (CSD), reference: 2344751. The unit cell parameters of the resolved form are a = 5.87820 Å, b = 9.5997 Å, and c = 45.0181 Å, and α = β = γ = 90° of the P212121 space group.

#### 2.6.2. Single-Crystal X-ray Diffraction (SC-XRD)

Single crystal X-ray diffraction data were acquired on a Rigaku Synergy-S system with a Dectris detector (XtaLab Synergy-S, Rigaku Corporation, Wroclaw, Poland) using Cu Kα radiation (λ = 1.54059 Å). The data were collected at 100 K. The structure was solved using SHELXT [44] and refined by least squares refinement using SHELXL [45] within OLEX-2 version 1.5 [46].

### 2.7. X-ray Photoelectron Spectroscopy (XPS)

#### 2.7.1. Powder Samples

X-Ray Photoelectron Spectroscopy was performed on tablets made of the Odanacatib samples. The 80 mg tablets, pressed at a load of 100 N, were made using the Gamlen press (Model GTP-1, Gamlen Tableting Ltd., UK). Following a relaxation period of at least 24 h, the surface chemistry of the tablets was analyzed using a Thermo Fisher K-Alpha+ that uses a Monochromated Al Ka Micro-focused X-ray source. A 5 mm^2^ area of each tablet was carefully cut using a scalpel and mounted on the sample holder using carbon tape. On each sample, a point of spot size of 400 µm was chosen for analysis. Following a quick survey to identify the elements in the sample, C1s, O1s, N1s, F1s, and S2p spectra were acquired at 20 eV pass energy. All spectra were Shirley background subtracted and fitted using a Gaussian/Lorentzian (25:75) mix. Fitting was carried out using the Avantage v5 software, using the minimum number of peaks required to minimize R-factor.

#### 2.7.2. Single Crystal

The stacked crystal structure has 3 distinct facets, as would a simplified rod-like structure. Each of these 3 facets was analyzed using XPS by carefully cleaving the crystal and mounting the desired face up on the sample holder using carbon tape. Measurements and analysis were carried out in a similar manner as for the tablets.

## 3. Results

### 3.1. Surface Area Using N_2_ Adsorption and IGC

**Figure 2:**
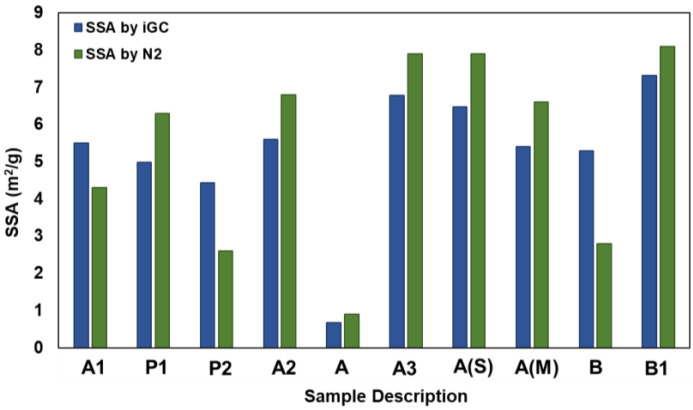
A comparison of the specific surface area of samples obtained via IGC and N_2_ sorption method.

Figure 2 shows the comparative SSA data obtained from N_2_ adsorption versus IGC. Both the N_2_ adsorption and octane isotherm measurements yield similar trends in the specific surface areas of the samples. Overall, the N_2_ BET showed higher values than the C8 IGC BET isotherm. This is expected to be due to the probe molecule size difference (C8>>N_2_) [47]. Among all the processed samples, sample A exhibited the lowest SSA (~0.7 m^2^/g) and sample B1 showed the highest SSA (~8 m^2^/g).

In general, unmilled samples A, B, and P2, with the exception of sample P1, showed a lower SSA compared to milled samples. As sample P1 is a fine powder with similar characteristics to the milled samples, its SSA aligns with the latter. However, a significantly higher difference in the SSA values measured for sample B was observed. Sample B exhibits a needle-shaped morphology with high aspect ratio. Thus, particle breakage of these API needles during the IGC sample preparation and analysis could have led to higher SSA. Similarly, the SSA for sample P2 obtained from IGC was 1.7 times higher than that from the N_2_ adsorption method. This was attributed to the extensive agglomeration observed and the differences in these agglomerated sample structures arising during IGC sample preparation and analysis. SEM images of Sample P1, Sample B, and Sample P2 can be found in Figure S1 in the Supplementary Information. Nevertheless, these findings suggest a consistent characterization of the material’s surface properties by these two analytical techniques. Such convergence in findings enhances the confidence in the material’s characterization and underscores the importance of using complementary techniques to gain a comprehensive understanding of its surface properties.

### 3.2. Surface Energy Heterogeneity of Processed APIs

**Figure 3:**
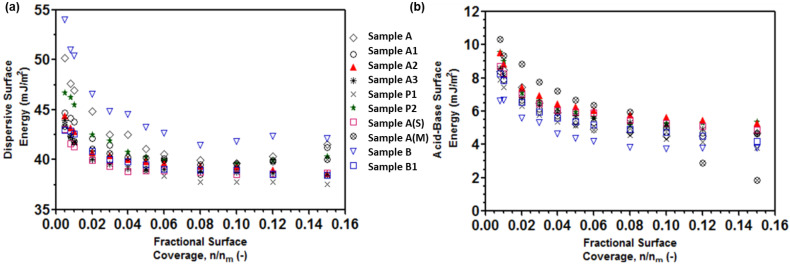
Dispersive (**a**) and acid-base (**b**) surface energy heterogeneity profiles of Odanacatib samples.

The dispersive (γ^d^) and acid–base (γ^AB^) surface energy heterogeneity profiles of the crystalline API samples are shown in Figure 3. The differences in dispersive and acid–base surface energies arise from the anisotropic nature of crystalline surfaces resulting from the exposure of various facets. This anisotropy and the resultant variations in the surface energy components observed for the API samples was postulated to be due to processing-induced morphological, surface-topographical, and chemical alterations. It is interesting to note that sample B exhibited the highest aspect ratio and span in the particle size distribution. Thus, for sample B, particle breakage of the thin needle crystals could have exposed new facets with higher γ^d^. Also, acetone:DIW-crystallized (A) and -precipitated crystals (P2) showed higher γ^d^. Overall, the samples exhibited a surface energy heterogeneity with γ^d^ trend as sample B (acetone:MTBE, high aspect ratio) > sample A (acetone:water, lowest SSA) > sample P2 (precipitation acetone:water).

On the other hand, sample B (highest AR) exhibited the lowest γ^AB^; however milling (sample B1) led to an increase in γ^AB^. Co-processed samples exhibited slightly higher γ^AB^ than without processing aid. Particularly, the MgSt coprocessed sample exhibited a very high γ^AB^ heterogeneity with high surface energies at low coverages, which dropped down dramatically at higher probe coverages (>0.08 p/p0). This could indicate the uneven distribution of MgSt across the API surface, leading to localized variations in surface energy, while some may form more uniform surface coverage. Consequently, there would be areas where the surface energy is high (due to more exposed active groups) and areas where it is low (due to MgSt coverage). Overall, the disparities observed in both the γ^d^ and γ^AB^ surface energies across the API samples point to distinct variations in the specific surface character like adhesion or chemical interactions induced due to processing. Moreover, considering the impact of these surface energies on the behavior of crystalline API, it can be inferred that γ^d^, which signifies the interparticle cohesive–adhesive interactions, is expected to influence powder mixing performance (CU). Similarly, γ^AB^, which elucidates the polar characteristics of surfaces, is anticipated to highlight surface wetting and dissolution performance (IDR). Detailed discussions on the implications and correlations of these factors are provided in the subsequent Section 4.

### 3.3. Surface Wettability as a Function of Storage Time

**Figure 4:**
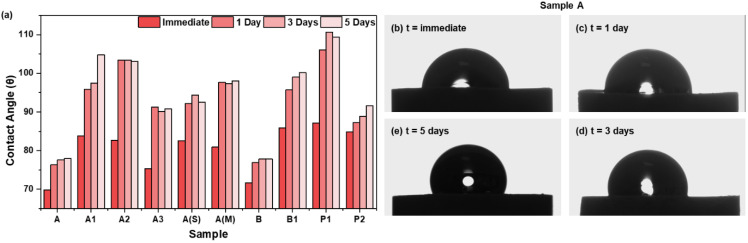
(**a**) Contact angle of Odanacatib tablets immediately after being pressed and after 1, 3, and 5 days of storage time. (**b**–**e**) Water droplet on the tablet immediately after compaction, and after 1 day, 3 days, and 5 days, respectively.

The contact angle measured on the API tablets revealed an initial (measured immediately after compaction) hydrophilic surface character, with contact angles < 90° for all the processed API samples (Figure 4). However, regarding storage, all the samples, except for sample A and sample B, showed an increase in the contact angle (dθ) values, exhibiting a more hydrophobic surface character with storage time. The dθ was also observed with these two samples, but not as significantly as with other tablet samples. This was hypothesized to be due to particle breakage of the high-aspect-ratio crystals during tablet compaction that led to the exposition of high-energy surfaces and, thus, higher surface hydrophilicity. The highest variation in dθ was observed with samples A1, A2, and P1, where the contact angle jumped from ~80° to ~110°.

The surface wettability of tablets may decrease over time as a consequence of stress relaxation behavior within the tablet matrix. Further measurements of tablet dimensions and porosity would be required to quantify the elastic recovery of the tablets for future investigations. However, during storage, it is expected that the initial stress within the tablet gradually relaxes. Contact angle is a function of surface chemistry, porosity, and roughness. These factors affecting the contact angle could undergo changes under storage conditions [48]. This decrease in surface wettability can be significant in pharmaceutical applications, as it may impact drug dissolution rates and ultimately affect the therapeutic effectiveness of the tablet. Thus, to maintain consistent drug release profiles, it is essential to consider the time-dependent changes in tablet surface wettability and develop strategies to mitigate any adverse effects.

### 3.4. PXRD

**Figure 5:**
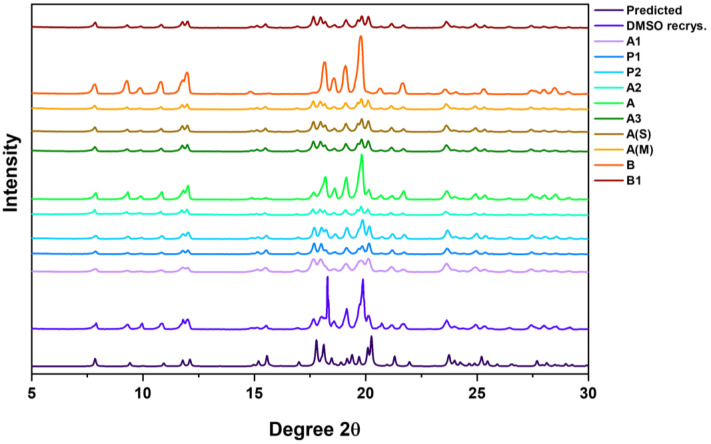
X-ray diffraction patterns of all the as-received samples and the crystals grown using DMSO, compared against the predicted powder patten.

The powder diffraction patterns of the as-received samples were confirmed to the be the same polymorph and are consistent with the PXRD pattern of the crystals obtained from DMSO (Figure 5). Although samples A and B have the same peak positions, the intensity of these peaks is relatively high and demonstrate smoother profiles compared to the other samples with shorter and noisy peaks. This was postulated to be due to the shape of the A and B particles. As these are the unmilled samples, the relatively large particle size could have led to preferred orientation as well as the scarcity of internal facets, as opposed to a milled sample, which may have resulted in smoother peaks. Regardless of the processing history and source of the crystalline samples, the same crystalline structure was observed.

### 3.5. XPS

#### 3.5.1. Single-Crystal Facet Analysis and Indexing

**Figure 6:**
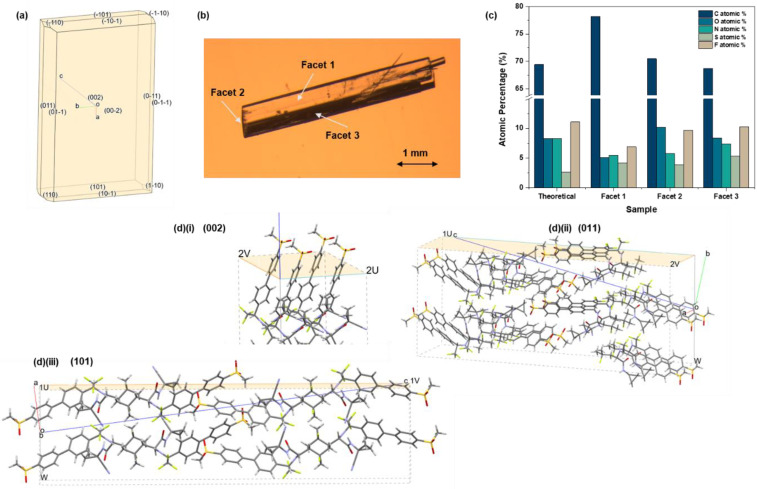
(**a**) Predicted BFDH morphology of Odanacatib, (**b**) ‘Stacked’ structure of single crystals grown in DMSO labelled with 3 major facets, (**c**) Atomic percentages of elements for facets 1, 2, and 3 obtained using XPS, (**d**(**i**–**iii**)) surfaces of facets (002), (011), (101) obtained using Mercury. Where a, b, and c are the crystallographic axis, U and V are vectors to define the size of the surface and W is the surface slab thickness factor.

The equilibrium Bravais Friedel Donnay and Harker (BFDH) morphology [49,50,51] of Odanacatib predicted using Mercury (Figure 6a), and the three major facets of the cuboidal habit were deduced to be from the [52] <011>, <101>, and <002> family of facets. The slip planes were also predicted using Mercury, based on the interplanar spacing and the hydrogen bonding interactions between the planes. Those marked (012) and (01-2) were identified as the slip planes, as they have the highest d-spacing and no interplanar hydrogen bonding, making them weakly interacting and highly likely to slip. The simulated powder diffraction pattern from the single crystal structure is consistent with the powder diffraction pattern of single crystals grown from DMSO. Meanwhile, the powder XRPD pattern of sample A1 (the starting material for the single crystal) shows an indication of preferential orientation. This indicates that the grown single crystal from DMSO has morphology that is in good agreement with its predicted counterpart, even though the final crystal shape demonstrated a high aspect ratio (Figure 6b). This is most likely to be due to the crystallization conditions.

XPS analysis was performed on the three main observable facets of the single crystals, arbitrarily labelled as facets 1, 2, and 3, as seen in Figure 6b. The elemental composition of the three facets is summarized in Figure 6c. Notably, the percentage atomic composition for each facet varies from the theoretical composition of the MK-0822 molecule. This is because each facet is formed by planes that slice through the crystal lattice at a unique angle. Hence, the surface chemical functionality on each facet differs based on the orientation of molecules along the plane of that facet (Figure 6d).

**Figure 7:**
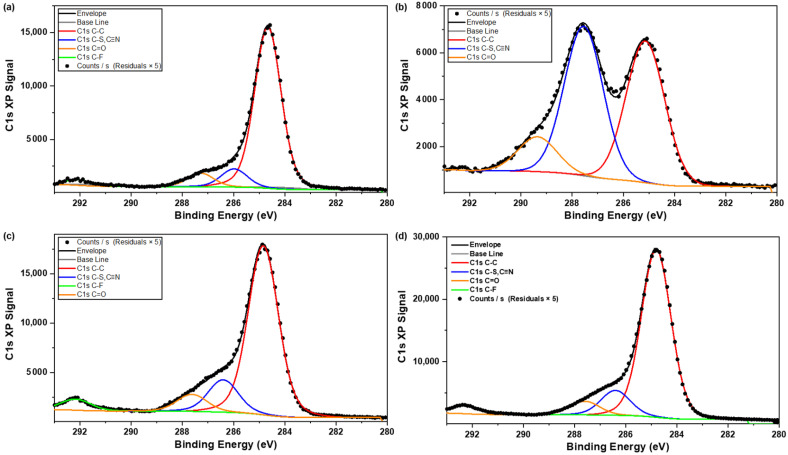
C1s spectra of (**a**) facet 1, (**b**) facet 2, and (**c**) facet 3 of Odanacatib crystal, and (**d**) C1s spectra of a tablet of Sample A1.

As carbon is the most abundant element and the spectra hold information about all the major functional groups of the molecule (C–C, C≡N, C–S, C=O and C–F), the peak-fitted C1s spectra of the three facets were utilized as a means to quantify the contribution of these functional groups (Figure 7a–c). Using the NIST XPS database, the C–C peak was labelled at 285 eV, C=O at 288 ± 1 eV, C–F at 292 eV, and a combined peak for the C≡N and C–S 287 ± 1 eV. The C1s spectra of a tablet of sample A1 is shown in Figure 7d and can be used as a representative C1s spectra for the powdered samples. The position of the peaks and the bonding environments of the crystal, as well as the powders, show good agreement.

#### 3.5.2. Powder Samples

**Figure 8:**
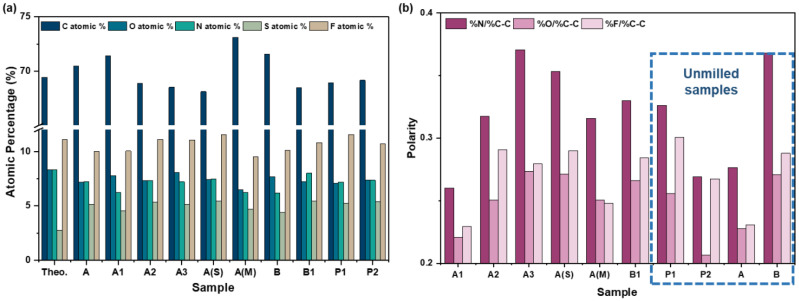
(**a**) Atomic composition and (**b**) calculated polarity of as-received samples after compaction into tablets.

The atomic composition of all the samples was obtained via analyzing the respective tablets under XPS (Figure 8).

Although the measured quantities were within an appreciable range of the theoretical atomic composition of the Odanacatib molecule, the exact composition varied by more than 2% between each sample, which is above the sensitivity of the instrument, and, hence, was presumed to be due to the processing history of each sample. The C1s, N1s, O1s, F1s, and S2p spectra were deconvoluted and peak-fitted with the bonding environments using the same method as for the single crystals, as detailed in Section 2.7. The Avantage software was used to convert the contribution of all the bonding environments across all the spectra into a percentage contribution for the sample, such that the total of all functional groups equaled 100%. Following this, the same functional groups were grouped together and their percentage compositions were summed to obtain the overall percentage contribution of the non-carbon elements across all the spectra. For example, C1s C=O, O1s =O, and O1s –O were added together to obtain the total %O. These were then divided by the non-polar C1s C–C composition to obtain the ratio of polar component to the non-polar component of the sample. This ratio is how ‘polarity’ of the sample is defined from this point forward. Sulfur was excluded from this analysis, as it was the least-abundant element across all samples, with its quantities being close to the sensitivity of the instrument. Hence, to avoid inaccurate interpretation of the sulfur data, it was excluded from the polarity calculations above.

## 4. Discussion

### 4.1. Correlations between Orthogonal Techniques

#### 4.1.1. Surface Energy vs. Polarity

**Figure 9:**
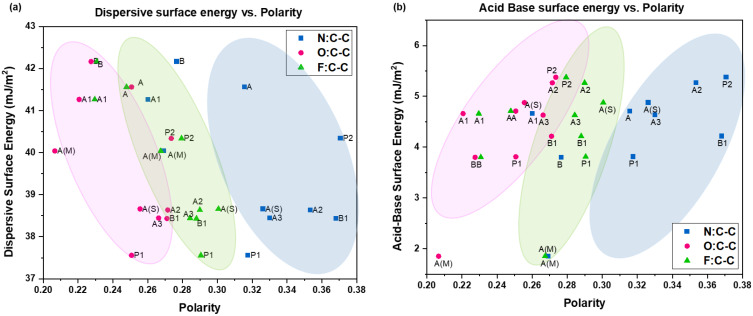
Correlations between sample polarity determined using XPS and (**a**) dispersive surface energy and (**b**) acid–base surface energy obtained using IGC.

Results from IGC were compared with those from XPS, with an attempt to discover viable correlations between surface chemistry and surface energy. From Figure 9, it can be seen that the acid–base surface energy has a strong positive correlation with the polarity of the sample with respect to all three elements. Intuitively, the opposite is true for the dispersive energy at 15% surface coverage where, as the amount of O, N, and F in the sample increase, the dispersive energy is seen to decrease. Correlations in both cases appear to become stronger with increasing surface coverage, with 15% surface coverage exhibiting the most robust trends. In Figure 9b, sample A(M) is seen to be a clear outlier from the trend, thus emphasizing the role of the additive (MgSt) in the variation of surface energy and surface chemistry.

#### 4.1.2. Processing History and Polarity

It was postulated that the milled samples will exhibit a higher content of polar components, as opposed to the unmilled samples—this chain of thought arising from the facet analysis of Odanacatib single crystals. As the crystals are rod-like/high-aspect-ratio, they are most likely to break perpendicularly to the longest facet [53,54], to expose facet 2 (Figure 6b). Therefore, post-milling, where these crystals would be exposed to constant breakage in the perpendicular direction, multiple crystalline particles exposing facet 2 would be produced. As the analysis in the above section highlights the high polarity of this facet in comparison to facet 1 and 3, milled samples would hence display a higher overall polarity. This was seen to hold true for samples A2, A3, A(S), A(M), and B1 (Figure 8b). Out of the unmilled samples, P1, P2, A, and B, only A and P2 samples exhibited a relatively low content of polar elements owing to the possibility that these samples were unbroken and, hence, did not expose high-energy facets.

However, samples P1, B, and A1 do not follow the aforementioned trends. In these cases, only considering the processing history of the samples is not sufficient—the particle shape and size also need to be taken into account. Figure S1 in the Supporting Information highlights the particle habit as observed via SEM [39] of Sample A, Sample B, and Sample P1. It can be seen that, even without undergoing milling, sample P1 is a fine powder. Hence, the small particle, resulting in more overall facets available for measurements, could result in a higher polarity. Moreover, the needle-like nature of sample B could have resulted in breakage during sample preparation, again resulting in an increased exposure of high-energy facets, causing it to be an outlier from the trend. Finally, as sample A1 is a milled version of sample A, which is a low-aspect-ratio crystal in comparison to sample B, milling sample A may not have resulted in extreme breakage and, hence, produced broken rods/block-like crystals. Thus, the XPS results for sample A and A1 show similar surface chemistries.

### 4.2. Correlations between Powder Properties and Performance Indicators

#### 4.2.1. Polarity vs. Intrinsic Dissolution Rate (IDR)

**Figure 10:**
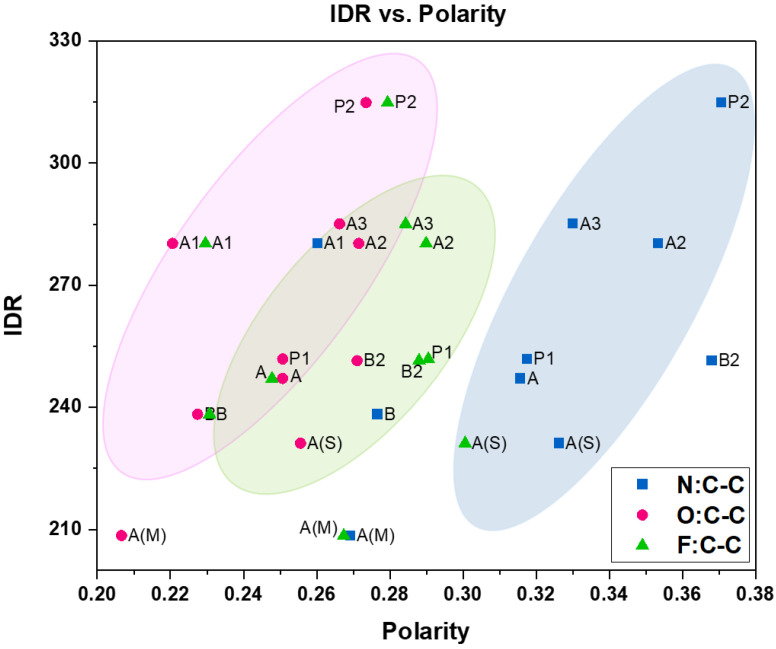
Correlation between intrinsic dissolution rate and polarity of Odanacatib samples.

The intrinsic dissolution rate (IDR) of the sample tablets in pure ethanol investigated by Wang et al. [38] was also compared to the sample polarity (Figure 10). Generally, samples with a higher content of O and N demonstrated a higher dissolution rate, which could be explained by the affinity of the polar bonds to the hydroxyl group in ethanol, resulting in faster dissolution. This trend was not observed for the fluorine composition of the samples.

#### 4.2.2. Intrinsic Dissolution Rate (IDR) vs. Acid–Base Surface Energy

**Figure 11:**
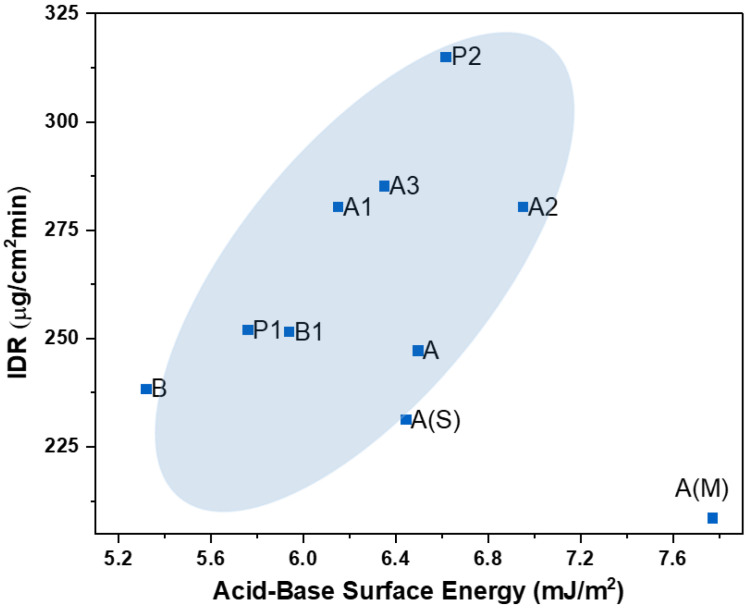
Correlation between intrinsic dissolution rate and acid–base surface energy.

Surface polar or non-polar characteristics can strongly influence the wetting and initial contact between a solid and a solvent during the dissolution process. In general, a trend of increase in the IDR with increasing surface polarity was observed with the available dataset (Figure 11). The acid–base component of surface energy (γ^AB^) represents the surface polar character and vice versa for the dispersive component of surface energy (γ^D^). Hence, with increasing γ^AB^, an increase in the IDR was seen with most of the crystalline APIs. An increased intrinsic dissolution rate with higher acid–base surface energy suggests that the API surface is more inclined to engage in polar interactions with the dissolution medium. This enhanced interaction promotes a faster dissolution process, as the solvent molecules can effectively penetrate and solvate the solid particles. The acid–base surface energy essentially facilitates the initial wetting and adhesion of the solid surface by the solvent, allowing for a quicker and more efficient breakdown of the solid material into solution. However, co-processed APIs, sample A(S) and A(M), were outliers for this correlation. In conclusion, the γ^AB^ can strongly influence dissolution of solids with a compatible solvent by promoting the wetting of the solid surface, leading to faster dissolution. This is especially relevant when considering the adhesion and spreading of liquid on a solid surface.

No such strong correlation was observed between IDR and γ^D^, as seen in Figure S2 in the Supplementary Information.

#### 4.2.3. Content Uniformity vs. Surface Energy

**Figure 12:**
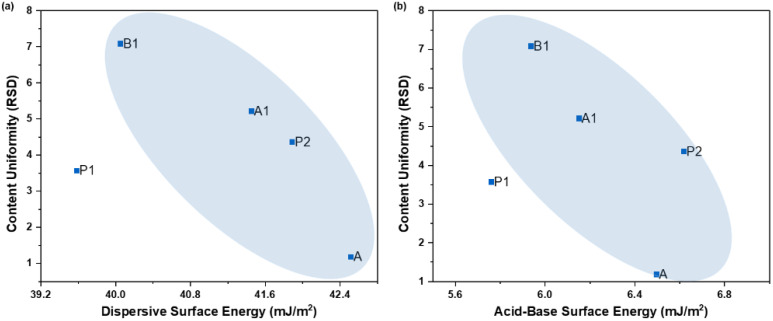
Correlation between content uniformity RSD values and (**a**) Ddspersive surface energy and (**b**) acid–base surface energy.

Among the other important factors, like particle size distribution and API concentration, surface energies and the content uniformity (CU), expressed in terms of RSD, for five tablet formulations (1% API, 27% MCC, 66% lactose monohydrate 5% crospovidone, and 1% MgSt) [38] with crystalline APIs were observed (Figure 12). That is, the higher the dispersive and acid–base components of surface energies, the higher the CU of the formulation. Thus, it can be said that surface energy can indirectly impact content uniformity through the interplay of cohesive–adhesive interactions between the API and the formulation excipients. Higher surface energies of the API particles would lead to higher propensity for such adhesive interactions during the formulation unit operations like powder mixing/blending and lubrication. Nevertheless, it is essential to understand that other processing operations like granulation, drying, and compression can equally impact the CU.

## 5. Conclusions

Results highlight the importance of considering crystal anisotropy and surface properties of materials as impacted by the crystallization and processing steps. Particle size and surface area could otherwise suggest comparability of materials, but, as demonstrated here, disparities in the preferred crystal faces can result in meaningful difference in surface properties that translate to bulk powder properties. The γ^d^ and γ^AB^ surface energies seem to be a reasonable predictor of content uniformity and intrinsic dissolution rate, respectively, owing to the potential differences in cohesive/adhesive and surface characteristics of these crystalline samples. The careful application of orthogonal characterization tools here also highlights important considerations when evaluating materials—including potential variability introduced by particle breakage during analysis and the importance to carefully condition all materials similarly so as to not introduce additional measurement system variability.

## Figures and Tables

**Table 1:** Particle size distribution and description of Odanacatib (MK-0822) samples.

Sample	d_50_ (µm)	Description
A	21.8	Heat cool in acetone:DIW parent lot for dry milling
A1	3.4	Spiral milled Sample A, annealed
A2	3.2	Spiral milled Sample A, annealed (2nd sample, different lot to assess PSD impact)
A3	4.9	Spiral milled Sample A, held at −8 °C after milling
A(S)	1.9	Spiral milled Sample A, with water soluble processing aid (1% SSF)
A(M)	2.6	Spiral milled Sample A, with water insoluble processing aid (1% MgSt)
B	15.8	MTBE:acetone recrystallization—modified solvent system to alter morphology and explore impact on breakage planes during spiral milling
B1	1.6	Spiral milled sample B
P1	2.7	Direct precipitation from DMF:DIW
P2	4.9	Direct precipitation from acetone:DIW

## Data Availability

The data presented in this study are available on request.

## Supplementary Materials

The following supporting information can be downloaded at: https://www.mdpi.com/article/10.3390/pharmaceutics16070883/s1, Figure S1: SEM images of samples (a) A, (b) A1, (c) B, (d) P1, and (e) P2; Figure S2: Correlation between intrinsic dissolution rate and dispersive surface energy.
