# Supplementary material for: Process-Induced Crystal Surface Anisotropy and the Impact on the Powder Properties of Odanacatib
Source: Pharmaceutics. 2024 Jun 30;16(7):883. doi: 10.3390/pharmaceutics16070883 (PMC11279451; doi:10.3390/pharmaceutics16070883)
Supplement: Supplementary file 1 [file pharmaceutics-16-00883-s001.zip › pharmaceutics-3024246-supplementary.pdf]

Process-Induced Crystal Surface Anisotropy and the Impact on the Powder Properties of Odanacatib

Isha Bade [1], Vikram Karde [1], Luke Schenck [2], Marina Solomos[2], Margaret Figus [3], Chienhung Chen [3], Stephanus Axnanda [3,*], Jerry Y. Y. Heng [1,*]

[1] Department of Chemical Engineering, South Kensington Campus, Imperial College London, London SW7 2AZ, UK; isha.bade16@imperial.ac.uk (I.B.); v.karde@imperial.ac.uk (V.K.)
[2] Oral Formulation Sciences, Merck & Co., Inc., Rahway, NJ 07065, USA; luke_schenck@merck.com (L.S.)
[3] Analytical Research & Development, Merck & Co., Inc., Rahway, NJ 07065, USA; margaret_figus@merck.com (M.F.); chien-hungbill.chen@merck.com (C.C.)
* Correspondence: stephanus.axnanda@merck.com (S.A.); jerry.heng@imperial.ac.uk (J.Y.Y.H.)

1. SEM images of Odanacatib samples

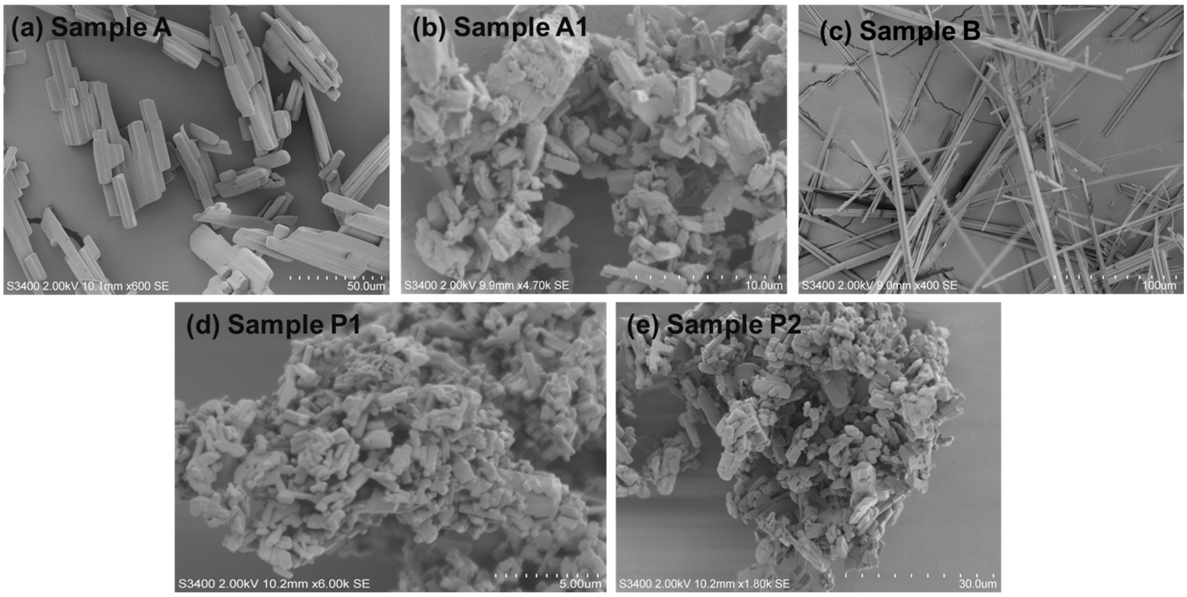

Figure S1 SEM images of samples (**a**) A, (**b**) A1, (**c**) B (**d**) P1 and (**e**) P2.

2. IDR vs. Dispersive surface energy

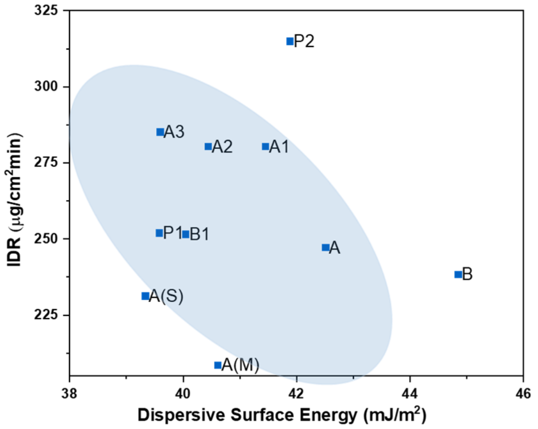

Figure S2 Correlation between Intrinsic Dissolution Rate and Dispersive surface energy.
